# Improving Construction Industrialization Practices from a Socio-Technical System Perspective: A Hong Kong Case

**DOI:** 10.3390/ijerph18179017

**Published:** 2021-08-26

**Authors:** Xin Jin, Geoffrey Q. P. Shen, E. M. A. C. Ekanayake

**Affiliations:** Department of Building and Real Estate, The Hong Kong Polytechnic University, Hung Hom, Kowloon, Hong Kong; verna.x.jin@connect.polyu.hk (X.J.); geoffrey.shen@polyu.edu.hk (G.Q.P.S.)

**Keywords:** Construction Industrialization, socio-technical system, social network analysis, Hong Kong

## Abstract

Construction Industrialization (CI) tends to improve industrial performance and contributes substantially towards global sustainability. Considering these merits, many countries and regions, including Hong Kong, have released policies to promote CI uptake. However, those policy interventions ignore the dynamic influence of stakeholders and technologies, which significantly influence the efficient management of CI. In response, this study aimed to objectively depict a real socio-technical system of CI uptake based on a representative case study in Hong Kong. Further, this study identified the critical issues associated with the CI uptake and proposed policy-related recommendations to overcome the key issues. In addition, this study proposed a novel approach based on two-mode social network analysis to facilitate the analysis from a socio-technical perspective. Theoretically, this depicts the interactions of construction industry stakeholders and artifacts within a dynamic, complex socio-technical environment, indicating a new stance for construction management. Finally, this research also provides valuable implications for the government to anticipate the impact of different CI policies on promoting its uptake within the complex socio-technical system.

## 1. Introduction

Construction Industrialization (CI) is an innovative and effective manufactory-based mode of construction [1]. As an alternative to the traditional onsite construction method, it has been progressively adopted throughout the world in recent decades [2]. Li et al. [3] also observe that: CI fulfils the triple bottom line of sustainability because (a) “leanness” of the processes improves economic sustainability; (b) reductions in resources utilization and enhanced reusability improve environmental sustainability; and (c) construction automation promotes social sustainability. The primary focus of CI is on manufacturing or factory-based production, which creates a controlled environment in the onsite assembly [4]. The superior performance of CI in saving production time, reducing waste, more effectively controlling quality, and reducing labor demand has made it popular for public housing projects in Hong Kong [5,6]. Its use in the residential sector has significantly contributed to the sustainability of Hong Kong’s construction industry [5]. Considering the superior performance of CI in housing developments and its significant contributions to sustainability, many countries and regions have released policies to promote CI uptake.

Due to the overwhelming demand for high quality, affordable housing in Hong Kong (HK) over recent years, the HK government has implemented compulsory regulations and incentive schemes to adopt CI. Recently, the greater use of CI was promoted in the Chief Executive’s 2017 Policy Address [7], which implemented new technologies to improve productivity and cost effectiveness, such as CI to build large-scale construction projects to enhance the level of construction automation. However, policy interventions for promoting the uptake of CI invariably focus on incentive schemes and compulsory requirements while ignoring the dynamic influence of stakeholders and technologies, both of which significantly influence the efficient management of CI. Furthermore, the industry observes the policy-related issues in adopting CI [8,9], such as the legal framework issues, lack of design codes and standards for prefabricated components, and lack of government incentives [8]. In addition, the uptake of new technologies is within a social-technical system, which is neither entirely a function of market demand nor a simple process of material/technology substitution; rather, it is mediated and influenced by the social attributes, structures, and dynamic interactions of “actants” [10]. Coordinating a socio-technical system in the construction industry is a complex task because of the project-based characteristics of the industry [5]. Specifically, each project establishes a temporary network of stakeholders from different organizations, involving diverse professions with different interests. Because stakeholders do not act independently, new practices and technologies are often resisted due to the risks associated with intricate lines of relationships [11]. Stakeholders and technologies mutually constitute a socio-technical system, with its performance depending on the behavior, interactions, and interdependencies of actants [12] (stakeholders and artifacts in this case).

Because policies relating to CI are adopted in complex social contexts in which multiple interactions among actants influence performance, it is vital to fully understand the social environment and examine the impact of CI policies on its uptake in such an environment. The socio-technical system approach can provide comprehensive insights into the realistic social backdrop of CI uptake, key issues, and the dynamic interactions among actants. Given the aforementioned research importance, and the absence of such research to date, this study objectively depicts a real socio-technical system of CI uptake based on a representative case study in Hong Kong.

First, this study identified the critical issues associated with the CI uptake in Hong Kong. Then, the study explored the critical actants (stakeholders and artifacts) and their critical attributes related to key CI issues. Finally, this study proposed policy-related recommendations for CI uptake by overcoming the identified key issues. The findings from the research provide a valuable reference for the policymakers when anticipating the influence of different CI policies on promoting its uptake within Hong Kong’s complex socio-technical system. The forthcoming sections respectively explicate the comprehensive literature review conducted, research methods adopted, details of the case study conducted, and data analysis and results, followed by a focused discussion and the conclusions drawn from this study.

## 2. Literature Review

**Figure 1:**
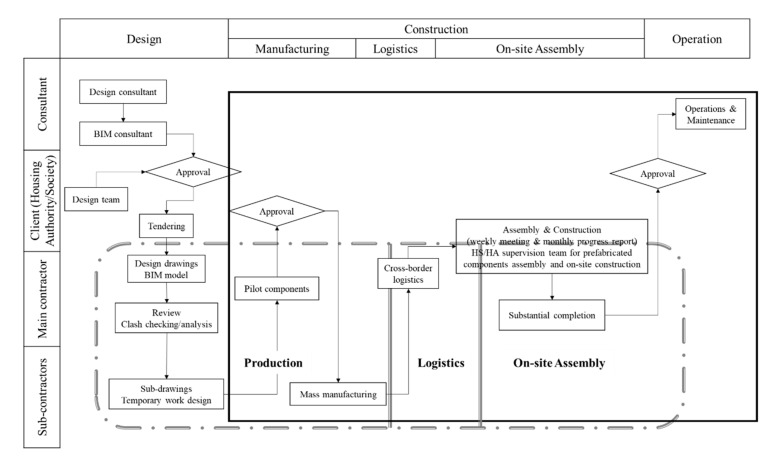
Flow chart of prefabricated housing production.

The socio-technical systems of the building construction sector are relatively complicated because the construction industry is different from, and even more complex, than other industries. This is mainly due to its decentralization and mobility [13], comparative undercapitalization, fragmentation, reliance on the sub-contract system, and influence of regular cycles of relatively high and low requirements [14]. The CI social subsystem includes clients, designers, manufacturers, contractors, and various suppliers distributed in different supply chain processes, from design, manufacturing, and transportation to onsite assembly. These stakeholders form a social network through formal (e.g., contract terms) or informal (e.g., trust among stakeholders) interactions [15]. Stakeholder relationships can significantly influence project performance; poor relationships can cause inferior results, such as cost overruns, time delays, and quality defects [16]. Therefore, the socio-technical subsystem should be controlled as a whole to coordinate the stakeholders and information. Considering the socio-technical subsystem of CI, Figure 1 shows the complex supply chain process and the stakeholders’ engagement in the general industrialized housing construction process in Hong Kong.

### 2.1. Issues Existing in the Application of CI

Given the significance of CI for improving construction quality and efficiency and strong environmental performance, many countries and regions are promoting CI in combination with relevant policies that focus on mandatory requirements and incentive schemes [17]. However, at the same time, some challenges and barriers to the application of CI cannot be ignored. Mao et al. [8] noted the top three obstacles that led to the failure of CI as lack of government regulations and incentives, high initial costs, and reliance on traditional construction methods. Through an SNA analysis, Gan et al. [18] indicated that the government and developers, as the principal stakeholders, play the most important role in establishing intensive cooperation with other core stakeholders to overcome barriers and promote the development of CI. This viewpoint is also supported by Luo et al. [5], who revealed that governmental policy change is a considerable risk for prefabricated building projects. For companies engaged in the prefab business, offsite facilities issues, shortage of skilled workers, financing issues, and capital investment were critical factors, and they were their particular concerns [19]. For industrialized buildings, market risk, onsite management risk, economic risk, and technical risk were identified as the significant barriers that inhibit CI implementation [20]. By comparison, for the public, the negative sentiment towards CI adoption is mainly due to the simple design, advanced technical level, safety issues, relatively high prices, and unemployment caused by insufficient skills [21]. The above-identified obstacles indicate that CI’s building performance, supportive policies, and the availability of the latest information of long-term significance are far from sufficient.

### 2.2. Actants in CI Uptake

The term “actant” was first mentioned by Latour [22] and refers to “anything that makes a difference in the situation”, which includes both human beings and other entities. Considering the characteristics of the construction field and combining the research objectives of this article, the actants comprise stakeholders and artifacts. A stakeholder is defined as any group or individual that may influence or be affected by achieving the objectives in CI projects [23]. In CI projects, without the stakeholders’ support, the organization would cease to exist; thus, they make a difference. An artifact is an entity viewed as a product of human conception or agency rather than an inherent element [24]. Ekbia [25] indicated that engineering artifacts begin with a set of ideas or “requirements” held by relevant stakeholders. Furthermore, this is typical for collaborative work in a building environment, where multiple stakeholders interact through a collection of diverse artifacts [26]. The collection can include digital project materials, engineering drawings, and other tangible artifacts, such as prefabricated components in this research. These artifacts should be shared and visible to the stakeholders [27]. As obtained from the current case study, the items in the collection may include documents such as master plans and design drawings, contracts to confirm prefabrication production, Building Information Modelling (BIM) models, and delivery/customer orders. Alternatively, the artifacts may contain technology and equipment related to prefabricated components, such as smart code tags, developed communication platform systems, trucks, and cranes.

### 2.3. Important Attributes of Actants

The authors identified important attributes of stakeholders and artifacts through a comprehensive literature review, as listed in Table 1. According to the classic theory recognized by many scholars [28,29,30,31], three attributes of stakeholders were utilized in this research: power to influence the project, urgency of the stakeholder’s claim on the project, and legitimacy of the stakeholder’s relationship to the project. Power is the ability of those who possess the authority to bring about the outcomes they desire; urgency means the degree to which a stakeholder’s claim calls for immediate attention; and legitimacy is “a generalized perception or assumption that the actions of an entity are desirable, proper, or appropriate within some socially constructed system of norms, values, beliefs, and definitions” [32].

The four attributes of the artifacts identified from the published articles are economics, user friendly, social acceptance, and environmental protection. With respect to CI, ‘economics’ considers the cost effectiveness of the artifacts, such as prefabricated façades. ‘User friendly’ indicates the artifacts are easy to use, reliable, and operational, such as the wearable radio-frequency identification (RFID) reader. ‘Social acceptance’ denotes the public acceptance of the artifacts, considering them as useful or convenient. ‘Environmental protection’ is relevant to sustainability and is extremely important to the development of society as a whole. All these identified stakeholders, artifacts, and attributes were then tested through a case study as described in the following sections of this paper. In addition, the existing issues relating to the uptake of CI and the possible solutions to overcome them were identified through a real-life case study.

## 3. Research Methods

This section presents research methods and analytical tools that were applied in this research. Through the comprehensive literature review, critical issues existing in Hong Kong for the application of CI, and the stakeholders, artifacts, and the attributes of CI, uptake were initially identified. According to the previous literature, a single representative case study is adequate to explore the embedded relationships of the actants under the social network analysis [5,39,40]. Thus, this study also adopted the single representative case study approach, and a case study was conducted using the data collection methods of document reviews, RFID tag scanning, onsite observation, and surveys with experts to provide real-life justifications of the literature findings and to generate the key research outcomes of this study. Current policy documents that influence the operation of CI uptake were reviewed, and project documents were examined to retrieve historical project information. Project documents relating to the CI application strategy and communication notes between stakeholders were collected to explore the internal interactions between the actants within the socio-technical systems, given that many Hong Kong construction firms record all their communication information through web-based communication systems that capture detailed information from emails, meetings, workshops, and other documentation formats during a project’s lifecycle.

An RFID tag is a machine-readable optical label that contains information about the object to which it is attached. To identify the interactions between stakeholders (usually site workers) and CI-related artifacts (such as materials, equipment, facilities, and tools) and recording process, time, and locations, an RFID tag system was used by attaching an RFID tag to each CI-related object and requiring workers to scan the tags, using their configured phones or RFID reader devices, whenever they started or finished the interactions each day in the selected project. Each RFID tag directed a request to a web server to record the artefact’s unique ID, the worker, time, and location (if necessary). The interactions between workers and artifacts were stored on a server to support the subsequent analysis.

Frequent site visits and onsite observations were conducted in the selected project through the following two channels: (1) the research team joined project team meetings at least eight times during the construction period (basically once per month) and observed the decision-making process, with particular focus on how stakeholders influence decisions; (2) the research team recorded the onsite construction process of industrialized elements with smart devices such as a video camera or smartphone for almost 3 hours.

In addition, structured surveys using semi-structured interviews were conducted to collect the personal attributes of stakeholders and the information regarding the key stages of the project. These included the key project stakeholders, namely the project manager, two assistant project managers, and project engineers with more than 15 years of experience in the CI projects, identified from the project documents. Five experienced professionals in academia were also included to provide their insights and valuable comments because they also had more than 20 years of industry experience. A total of nine experts participated in the interviews (the relevant details can be found in Table S1). In previous research attempts, the researchers adopted the SNA method by interviewing three experts [5] or seven respondents [41] to conduct the data analysis. Therefore, the number of respondents employed in this study was considered to be adequate to generate significant results, given the higher number of interviewees and their relevance to the study. Interviews with stakeholders focused mostly on three aspects that were difficult to identify from other methods: stakeholder informal social networks; the opinions of manufacturers, workers, and managers on efficiency, safety, and convenience regarding producing, transporting, and installing industrialized elements; and the opinions of the stakeholders regarding current CI-related policies and their desire for more promotional policies.

After the data collection process, data analysis was conducted. Coding of the gathered information and social network analysis were undertaken in this stage. Considering that the survey data was predominately numerical values and that the amount was manageable, data were coded manually with an Excel spreadsheet to show stakeholder attributes and social structure. A 0-1 matrix was developed to indicate the relationship between the stakeholders and the related CI artifacts, in which 0 represents no connection between the two entities, and 1 shows a correlation between them. For example, the RFID tag design must be approved by the Hong Kong Housing Society (HKHS) before it can be used in this project; thus, because the tag and the HKHS are relevant to each other, the number should be 1 at the intersection of their forms. The truck trailer and gantry crane used to help transport the prefabricated façades are managed by the manufacturer; because they have no connection with the HKHS, the number should be 0. The detailed information about the matrixes of these three stages can be found in Table S1.

Social network analysis (SNA) was applied in the stakeholder analysis of this research project. The nodes in the network were defined as stakeholders and/or artifacts, and the links as the relationships among them. SNA in stakeholder analysis provided the relationship structures of stakeholders, which is shown in a graph of the network [42]. For example, Liang et al. [39] adopted SNA to analyze the interactions between critical success factors and stakeholders from a stakeholder perspective in green retrofits. The graph also shows differently shaped nodes to represent the type of stakeholders, and the width of links describes the tightness of relationships, which cannot be captured directly from the original data. All coded information was imported into a social network analytic tool (supported by NetMiner software (CYRAM company, Gyeonggi-do, South Korea), generating numerous metrics, including all standard and specialized metrics for multi-mode social networks. NetMiner, a software package that features network data transformation, statistics, and visualization, allows authors to explore their network data visually and interactively, helping them to detect underlying patterns and demonstrate the network structures and strength of relationships among the categories [5,40]. At each measurement point, NetMiner shows the number and the type of link between nodes (stakeholders and artifacts) [43].

Critical actants were identified by calculating an important network analysis indicator, i.e., centrality, which revealed the structural configurations of the network; specifically, centrality identifies the most significant nodes in a network [39]. Although some direct relationships can be observed from the original data, such as whether a stakeholder is relevant to an artifact, the critical actants from a range of data and the whole network structure are hidden unless some SNA tools are applied. Moreover, some quantitative analysis in the SNA, such as measuring centrality values, ensures the conclusions are more scientific and reliable [5]. The outcome of this step determined the critical nodes and relations that have a considerable impact on the complexity of the socio-technical network [40]. The above indicator can prioritize the significance of critical actants, and their evolution trajectories throughout the system can be observed.

**Figure 2:**
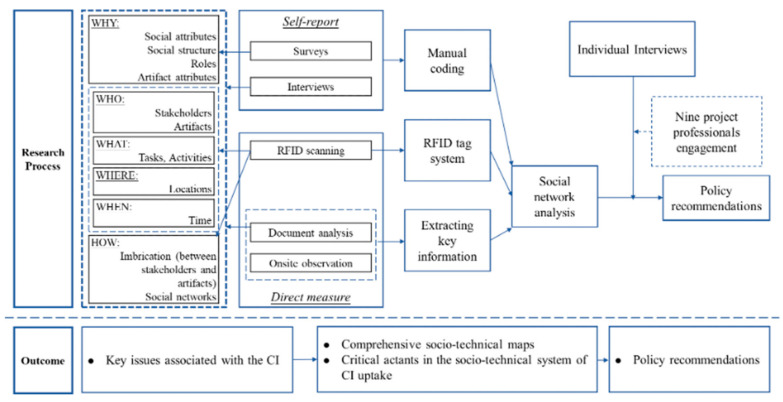
Integrated methods in the case study.

Socio-technical scenarios were then applied to provide valuable implications for the government to understand the impact of different CI policies on promoting its uptake within complex socio-technical systems. This project followed the research framework illustrated in Figure 2; multiple innovative methodologies were applied to generate relevant research outcomes.

### Case Study Details

**Figure 3:**
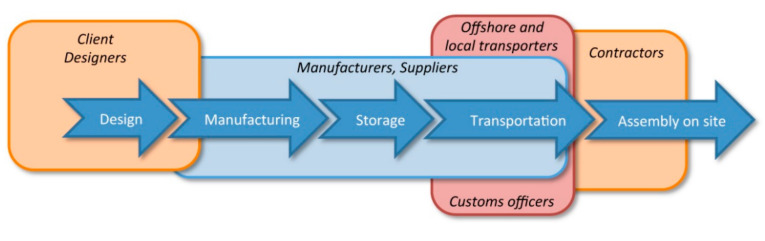
Offshore prefabrication housing processes.

The case project selected was a new prefabricated housing project owned by the HKHS, which was tracked continuously by the authors over the data collection period of nine months from December 2018 to August 2019. The HKHS has always been deemed a devoted organization, providing quality housing for the people of Hong Kong. This residential project developed by the HKHS is a typical public house using prefabricated components and is similar to most public housing buildings in terms of height, floor plan, structure type, and assembly cycle; thus, it is a representative CI building. Prefabricated façades were used in this project, which consisted of nine different kinds of modules to form 26 different types of façades to construct a 33-story residential tower. As the project client, HKHS coordinated and monitored the project quality, cost, and progress, from the inception stage until substantial project completion. Generally, the offshore prefabrication housing processes follow the procedures shown in Figure 3, which makes it possible to exploit the materials and labor force in Mainland China at an affordable price. This process enabled capturing the required information with prompt communication, observation, and monitoring. This pilot project identified the critical actants in this socio-technical system, and the visual comprehensive socio-technical network maps for CI uptake were developed as presented in the following sections.

## 4. Results and Discussion

### 4.1. Comprehensive Maps of the Socio-Technical System of CI Uptake

In the graph of SNA regarding the CI pilot construction project, nodes represent different stakeholders and artifacts identified in the previous step, and links display the relationship between them. Here, the matrix was used as an input because there were two separate entities, whereas a two-mode network was established to identify the relationship between stakeholders and artifacts. NetMiner was selected to form a visual comprehensive socio-technical network map. In addition to nodes and links, additional information can be illustrated in the output. For each stage, an independent analysis was conducted. Conceptually, when focusing on a two-mode graph, degree centrality is defined as the number of connection impacts on a node [30,44], which can be simply calculated by the portion of nodes adjacent to each node [45]. The main node and sub-node degree centrality vectors in production, transportation, and onsite assembly stages are presented in Table 2.

**Figure 4:**
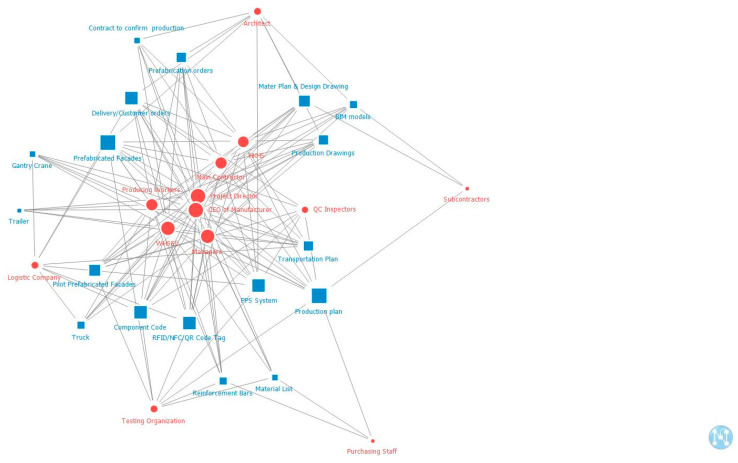
Two-mode degree centrality of stakeholders and artifacts at the production stage.

**Figure 5:**
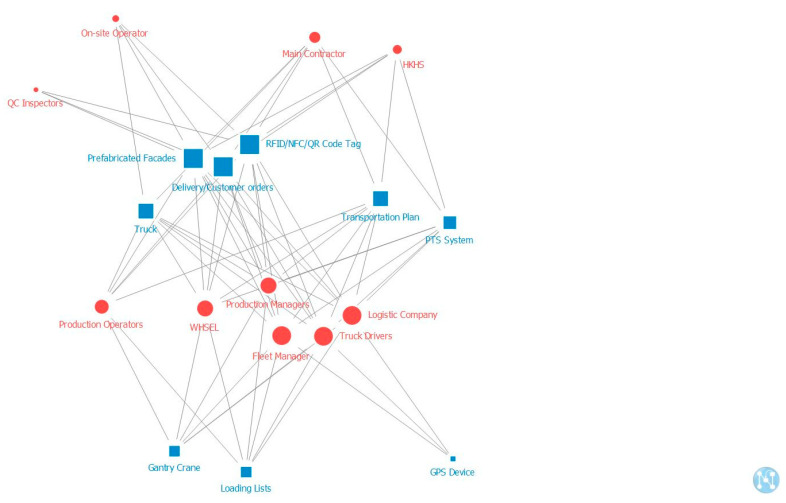
Two-mode degree centrality of stakeholders and artifacts at the transportation stage.

**Figure 6:**
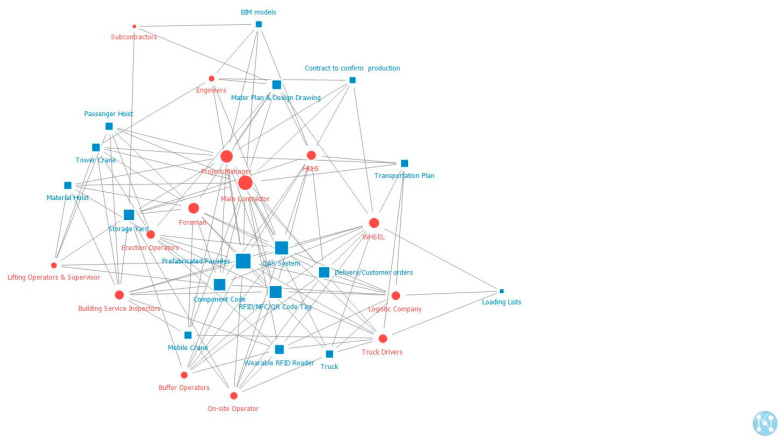
Two-mode degree centrality of stakeholders and artifacts at the onsite assembly stage.

Figure 4, Figure 5 and Figure 6 present the “two-mode degree centrality” networks of stakeholders and artifacts in production, transportation, and onsite assembly stages. Degree centrality is defined as the link that a node shares directly with other nodes and is commonly used for the structural importance of nodes because it focuses on the local structure in which a particular node is embedded [42]. In this study, the degree centrality of a stakeholder is the sum of the artifacts it is affiliated with, and the degree centrality of an artifact is the number of stakeholders affiliated with it. The shapes (such as round and square) and colors (blue and red) show the types of stakeholders and artifacts, whereas the length and width of links indicate the strength of the relationship. The red circles represent all the stakeholders, while the blue squares represent all the artifacts. If the size is bigger and closer to the center, the stakeholder or the artifact is more important to the whole network. The node with a high degree of centrality has direct weighted links to other nodes, greatly influencing its neighbors. The interpretation of degree centrality is that stakeholders are critical because of their influence level or the number of interactions they have. In addition, artifacts are notable because of their membership size.

### 4.2. Critical Actants in the Socio-Technical System of CI Uptake and Their Dynamic Evolution in the Project Lifecycle

Based on these comprehensive maps, the most significant stakeholders and artifacts in the network were identified. Previous studies have typically identified the top three or five elements of the indicators as the key factors because they mainly affected the network’s complexity and structure [5,46,47]. Similarly, this study also identified the top five actants in the ranking list as the critical/key stakeholders and artifacts. The top five critical stakeholders and artifacts at each stage are listed in Table 3. In all three stages, the most critical artifact is the prefabricated façade. However, at the production stage, the most critical stakeholder is the prefabrication manufacturer; at the transportation stage, the most critical stakeholder is the fleet manager; and at the onsite assembly stage, the most important stakeholder is the main contractor.

In terms of the critical stakeholders, at the production stage, the manufacturer is responsible for the overall control of the prefabrication workflows [48]. In addition, the manufacturer needs to respond actively and promptly to frequent revisions of the master program by the main contractor [5]. The timely information of the manufacturer is conducive to effective information sharing throughout the supply chain. At the transportation stage, the fleet manager controls the logistics of prefabricated components from the manufacturer to the assembly site, which mainly involves the cross-border logistics between mainland China and Hong Kong customs. To meet the requirements of safety, quality, efficiency, punctuality, reliability, and flexibility of transportation services, while reducing costs and empty mileage, the demands for efficient fleet management are increasing, especially in prefabrication transportation [49]. During the onsite assembly stage, the main contractor’s responsibilities are particularly prominent, as the main organizer and coordinator of the building project who is responsible for formulating the master program and scheduling milestones [5]. The main contractor is at the core of improving communication efficiency between project stakeholders [50]. As Gan et al. [18] indicated, a contractor is a core stakeholder who needs to establish intensive collaborative relationships with other core stakeholders to facilitate the smooth delivery of the entire prefabricated project.

Regarding the critical artifacts, in prefabrication housing, the prefabricated components (facades in this case project) are a major product and an important relation between stakeholders for a prefabrication supply chain. In addition, the application of information and communication technologies, such as RFID/NFC/QR code tags, can facilitate services, tools, and mechanisms for different stakeholders in three stages, increasing the success of daily operations and decision making across prefabricated housing project management to ensure timely project delivery [50]. This viewpoint was also supported by the project managers and professionals who participated in the interviews. They explained that these information tracking technologies and communication platforms (such as the OAS system) ably assisted timely information sharing throughout the supply chain and facilitated the orderly installation of the prefabricated components in the project, contributing to the smooth progress of the overall project schedule. In the production stage, delivery/customer orders and production plans are important because they are related to ensuring the entire component production line can proceed smoothly as required. Similarly, delivery/customer orders and transportation plans are significant in the transportation stage. In the onsite assembly stage, the component code is another critical artifact because relevant stakeholders can locate the related components or production lines in time in the unlikely event of an error.

During the survey within the case study, the correlation between stakeholders and attributes, and the correlation between artifacts and attributes, were graded. The grading criteria range from 1 to 5. Specifically, the number 1 indicates completely irrelevant, 2 indicates irrelevant, 3 indicates neutral, 4 denotes relevant, and 5 represents highly relevant. For instance, if the manager thought that attribute power is highly relevant to the main contractor, 5 would be displayed at their cross point. After collecting these assessment forms from the survey, the authors added scores for each attribute under each stage and generated the sum of each attribute for stakeholders and artifacts. The larger the total, the more important the attribute. The attribute corresponding to the maximum number is the most important attribute. The critical attributes of critical stakeholders and artifacts at each stage could then be determined following this principle. To display the results intuitively and conveniently, Table 4 summarizes the ranking of the attributes based on their importance.

From the above table, several significant conclusions can be drawn. At all three stages, legitimacy is the most important attribute of stakeholders, indicating that support from the government is necessary to promote different CI policies for its uptake. By comparison, at the first two stages of production and transportation, the most critical attribute of artifacts is social acceptance, whereas user friendly is the most important attribute of artifacts at the onsite assembly stage. This result shows that social acceptability of the policies should be prioritized; however, for actual onsite operations, workers always pay more attention to the user-friendly artifacts in terms of whether CI products or the equipment is easy to use, reliable, and operational.

### 4.3. Key Issues Existing in Hong Kong Regarding the Application of CI

Based on the literature review, onsite observations during the construction period, and nine expert interviews with relevant project stakeholders (project managers and engineers) of the selected case study, the following key issues existing in Hong Kong regarding the application of CI were identified:

A. Complex cross-border transportation process;
B. Low innovations in prefabrication techniques;
C. High initial cost;
D. Lack of preferential policy;
E. Low standardization;
F. Lack of management practices and experiences;
G. Non-timely communication between the parties.

In addition, the relevant policy recommendations are made based on the case study findings at the end of this section.

A. Complex cross-border transportation process

A complex cross-border transportation process and related issues are reflected in transportation constraints, which is a unique feature of Hong Kong that is different from other locations. Land resources available in Hong Kong are limited, and the suppliers have established their fabrication yards around the Pearl River Delta area because it is economical due to the lower cost of labor and land [5]. Prefabricated elements, such as precast concrete panels, precast façades, precast staircases, panel wall partitions, and semi-precast slabs, are relatively large and typically very heavy. Hence, based on the pilot project considered in this study, only a very small number of prefabricated façades (5–7) can be delivered by a heavy truck at one time. During the transportation of prefabricated components, not only additional costs will be incurred, but also greater time consumption will be required [51]. Automated data collection techniques are currently popular during the cross-border transportation process [52]. To further reduce the implementation cost of data collection, ensure that real-time information in the transportation process is adequate, and enable effective sharing of information among all involved stakeholders [49], some relationship-based data exchange platforms have been proposed (such as an RFID-enabled BIM platform) [50], in addition to some of the latest information tracking technology applications (such as IoT, RFID, QR code, and NFC) [53]. For precast concrete construction in Hong Kong, logistics usually consist of two procedures: cross-border transportation from the factory in Mainland China to the staging zone in Hong Kong (Logistics A), and local transportation from the staging zone to the construction site (Logistics B) [5]. To a large extent, the cost, timing, and progress of construction rely on the logistics of prefabricated module delivery. In this project, it took nearly two days to transfer the prefabricated façades from the factory to the laydown yard, and it took half a day to complete Logistics A, including the customs clearance time. Complex cross-border transportation processes mainly affect transportation and onsite assembly stages. In these two stages, the critical stakeholders related to this issue are the fleet manager, logistic company, truck driver, and main contractor, and the critical artifact pertaining to this issue is the truck. The most important attribute of stakeholders in transportation and onsite assembly stages is legitimacy. The critical attribute of artifacts at the transportation stage is social acceptance. A large amount of effort has been made in terms of both theoretical and technical aspects to promote the operation and management of fleet and logistics management in various fields [49], such as an Internet of Things (IoT)-enabled smart BIM platform [53].

B. Low innovations of prefabrication techniques

The core of improving the construction industry’s productivity lies in technological innovations [54]. The government has always attached great importance to promoting innovative building technologies [53]. The effectiveness of promoting CI adoption largely depends on addressing the technical issues. Additionally, several important factors deserve attention, including poor manufacturing capability and insufficient maturity of techniques used at the detailed design stage [55]. The detailed design is a multi-disciplinary process that includes assembly design and analysis, mold design, and piece and connection design [56]. The role of detailed design is to transform construction drawings into assembly drawings. This process is time consuming because it is based on two-dimensional computer-aided design (CAD) drawings. With the development of three-dimensional (3D) modelling software and BIM technology, the way building information is represented and managed has the potential to be revolutionized [57]. However, data interoperability between the software (e.g., Autodesk Revit, Tekla Structures, ArchiCAD, and GraphiSoft) [58] has not yet been achieved. Applicability of Just-In-Time (JIT) tools also requires technological innovations, which save construction time and enhance productivity [59]; however, there are many obstacles to JIT application that must be paid greater attention by construction organizations. Innovations in prefabrication techniques affect the production, transportation, and onsite assembly stages. In each of these three stages, the critical stakeholders related to this issue are the chief executive officer (CEO) of the prefabrication manufacturer, production project director, production managers, production workers, main contractor, project manager, agency (HA/HS), foreman, fleet manager, truck driver, and, in particular, the decision makers. The dominant attribute of stakeholders at the production, transportation, and onsite assembly stages regarding this issue is legitimacy. In the first two stages, production and transportation, the most critical attribute of artifacts is social acceptance, whereas user friendly is the most important attribute of artifacts in the onsite assembly stage.

C. High initial cost

A critical obstacle to the use of prefabricated construction is the associated high initial cost [8,60], which includes the initial investment (e.g., the costs of transportation and setting up prefabrication yards), the rent of high-quality hoist equipment for vertical transportation onsite, extra expenses related to labor training, and higher wages for skilled labor [61]. Tradesmen engaged in the construction industry have previously expected financial support from the government to balance the high initial cost [62]. The high initial cost mainly affects the production and onsite assembly stages. In these two stages, the critical stakeholders related to this issue are the prefabrication manufacturer, production workers, main contractor, project manager, agency (HA/HS), and foreman. The most important attribute of stakeholders in the production and onsite assembly stages is legitimacy. The critical attribute of artifacts in the production stage is social acceptance, whereas in the onsite assembly stage, it is user friendly.

D. Lack of preferential policy

The inadequacy of government incentives is a key issue that hinders the large-scale application of CI [63]. The main challenge identified in this respect is the lack of preferential policy [64]. From the aspect of incentives, multiple monetary incentive schemes like Joint Practice Note 1 confines GFA exemption to 8% cap for projects using prefabricated external walls [65]. However, this measure should probably be improved as time goes by, such as increasing the proportion of exemptions or expanding the scope of adoption of precast components. While the CI practitioners always expect more preferential policies to support the development of the construction industry [64], the government’s positive response could stimulate an increasing number of contractors to invest in prefabrication housing production. According to Luo et al. [20], preferential policies, including improving income tax incentives and subsidy provisions for CI, would generate a high positive impact on CI promotion. Preferential policies within Hong Kong mainly affect the onsite assembly stage. At this stage, the critical stakeholders related to this issue are the prefabrication manufacturer, main contractor, project manager, agency (HA/HS), and foreman. The most important attribute of stakeholders at the onsite assembly stage is legitimacy. The critical attribute of artifacts at the onsite assembly stage is user friendly.

E. Low standardization

Low standardization significantly affects the adoption of offsite construction [66]. It causes severe compatibility problems, especially when multiple manufacturers are involved in a CI project. Moreover, incompatibility has been a serious issue in implementing integrated, prefabricated façade development [67]. This is mainly attributed to the lack of peremptory industry norms for CI, which has been treated as a cornerstone of the overall success of adopting CI [68]. Without a national standard, most construction components are not standardized, making it hard to design a prefabricated building. Inappropriate design codes and standards for industrialized buildings is another reason for low standardization [20]. Although the government issued a code of practice for precast concrete construction in 2003, which was updated in 2016 [69], more universal mandatory standards should be promoted. This also emphasizes the government’s leading role in promoting CI by issuing and enforcing adequate policies and regulations. Low standardization mainly affects the production and onsite assembly stages. In these two stages, the critical stakeholders related to this issue are the prefabrication manufacturer, production workers, main contractor, project manager, agency (HA/HS), and foreman. The most important attribute of stakeholders in the production and onsite assembly stages is legitimacy. The critical attribute of artifacts in the production stage is social acceptance, whereas in the onsite assembly stage, it is user friendly.

F. Lack of management practice and experience

In following up the project, the authors found that prefabrication was still technologically unfamiliar to some local contractors. Most of these may have experienced projects using a few types of prefabricated components, such as precast slabs, façades, and staircases, but had less experience in more complex prefabrication systems that could be more complicated in arrangement and installation. With the experience gained, these contractors may be able to arrange the installation and handle the collaboration between prefabrication and onsite concreting components more effectively [36]. The project professionals mentioned that, for newcomers to the construction industry or ordinary workers, lacking knowledge and expertise of CI is a common phenomenon. The manufacturer’s experience of CI would be invaluable for achieving JIT and enhancing overall building efficiency [70]. The project manager’s ability to solve problems and cope with onsite management risks can be further improved through training. Lacking management practice and experience affects the production, transportation, and onsite assembly stages [20,71]. In each of these three stages, the critical stakeholders related to this issue are the CEO of the prefabricated components manufacturer, production project director, production managers, production workers, main contractor, project manager, agency (HA/HS), foreman, fleet manager, the truck driver, and, in particular, those involved in decision making. The domain attribute of stakeholders in the production, transportation, and onsite assembly stages is legitimacy. In the first two stages of production and transportation, the most critical attribute of artifacts is social acceptance, whereas user friendly is the most important attribute of artifacts in the onsite assembly stage.

G. Non-timely communication between the parties

Severe inconsistency between production, transportation, and onsite assembly shows poor communication among stakeholders, which is revealed as one of the root causes of poor supply chain management and low adoption of CI [5]. Poor interactions between stakeholders may be due to ineffective communication [72]. Progress updates and changes were exchanged mainly by email, WhatsApp, and hard copies of project documents, resulting in weak coordination between the upstream production of precast components and their downstream demand. Poor communication between stakeholders affects the production, transportation, and onsite assembly stages [73,74]. In these three stages, the critical stakeholders related to this issue are the CEO of the manufacturer, production project director, production managers, production workers, main contractor, project manager, agency (HA/HS), foreman, fleet manager, the truck driver, and, in particular, those involved with decision making. The main attribute of stakeholders in the production, transportation, and onsite assembly stages is legitimacy. In the first two stages of production and transportation, the most critical attribute of artifacts is social acceptance, whereas user friendly is the most important attribute of artifacts in the onsite assembly stage.

## 5. Policy Implications and Recommendations

Because policies are adopted in complex social contexts in which multiple interactions among stakeholders influence their performance, it is vital to fully understand the social environment and examine the impact of CI policies on its uptake. The socio-technical systems approach provides comprehensive insights into the realistic social backdrop of CI uptake and the dynamic interactions among stakeholders. From the research findings, legitimacy is the most important attribute of stakeholders, indicating that support from the government is necessary to promote different CI policies for its uptake.

The key issues reflect the limitations of temporary organizations, which are the features of construction projects, particularly the network associations among different actants. Such relationships reveal the uptake of CI over time in the complex socio-technical systems of the construction sector. The construction sector’s CI drivers (usually government, clients, and industrialization technology suppliers) are pressured to develop useful measures to effectively tackle these problems. In response to the above key issues, the same nine experts were again interviewed to discuss the policy implications and recommendations that could promote CI uptake in Hong Kong. Concerning the key issues identified as A to G above, the authors provide the following recommendations based on the experts’ interviews.

To address issue A (complex cross-border transportation process), the government should prioritize or privilege specific logistic companies in charge of transporting prefabricated components through the complex cross-border transportation process. This will enhance the efficiency of customs clearance, so that, the time taken to cross the border between Mainland China and Hong Kong is expected to be shortened from 40 minutes to 20 minutes. To address issue B (low innovations in prefabrication techniques), prefabrication techniques, use of new technologies, the precast rate, and adoption of different forms of precast components should be enhanced. Under current policies, the precast rate of public housing is around 30%, with precast components restricted to slabs, partitions, façades, beams, staircases, and bathrooms. Increasing the rate of precast use for public housing would promote CI application while breaking the limits of the scope of prefabricated components’ use and upgrading the commonly used prefabs to MiC through technological innovations, would also be helpful. In addition, more efforts should be on technical policies, such as promoting the use of information technologies including RFID, Quick Response (QR) codes, and Near Field Communication (NFC). Efficient supervision systems should also be promoted vigorously to record all processes of prefabricated components effectively [75], thus improving JIT and productivity by maintaining better control over the quality of construction.

To address issues C and D (high initial cost and lack of preferential policy, respectively), the government should encourage enterprises to develop prefabricated component factories under promotional policies that provide tax deductions, subsidies, fund rewards, or even interest-free finance to contractors who wish to purchase prefabrication plants; and open a preferential “green channel” for fast administrative approval of CI projects. To address issue E (low standardization), refining and enhancing policies associated with precast construction standards in both public and private sectors should be a priority for Hong Kong. Design guides, new codes, and standards for precast concrete construction should be developed and/or revised in line with the standards of Mainland China as appropriate, and they should be released as soon as they become available.

To address issue F (lack of management practices and experiences), training and education programs are proposed to explore the positive impacts of increasing stakeholders’ skills and willingness to adopt CI. Under managerial and educational policies, supportive training programs should be considered, and cooperation among research institutions, universities, and companies (setting up research funds) should be initiated so that newcomers to the construction industry would be more familiar with the CI concept and application. CI requires that practitioners be highly skilled [76] and have proper knowledge of industrialized building system operations; the provision of suitable training programs led by the government (in collaboration with the Hong Kong Construction Industry Council) is therefore considered an essential measure. For example, the government could offer consciousness forming and skill upgrading programs to private sector institutions that would encourage them to support the educational aspects of CI.

To address issue G (non-timely communication between the parties), advanced information technologies to monitor prefabricated modules on a real-time basis should be initiated to offset the poor communication between stakeholders. The pilot project of HKHS illustrated how the RBIMP (RFID-Enabled BIM Platform) and related technologies work in a real-life construction project, tested their functionalities, demonstrated their benefits and potentials, explored their boundaries for further improvements, and suggested improved agendas for future projects. Further, the government should expand funding for information technological innovations, such as the RBIMP, which could improve effective and timely communication between stakeholders.

## 6. Conclusions

CI is an innovative and effective factory-based construction method, which has been used in public housing projects for many decades in Hong Kong and contributes significantly to residential development. The superior performance of CI in saving production time, reducing waste, improving quality, and reducing labor demand makes it a suitable solution for Hong Kong to achieve performance enhancement and sustainable development. Many policy interventions for promoting CI uptake invariably focus on incentive schemes and compulsory requirements while ignoring the dynamic influence of the actants (stakeholders and artifacts). Notably, this influence significantly affects the management efficiency of CI. The socio-technical systems approach provides comprehensive insights into the realistic social backdrop of CI uptake and the dynamic interactions among stakeholders.

After collecting the relevant information, this study developed comprehensive maps of the socio-technical system of CI uptake considering the production, transportation, and onsite assembly stages using NetMiner software. From these maps, the authors identified the critical stakeholders and artifacts in the socio-technical system of CI uptake, in addition to their dynamic evolution in a project lifecycle. In all three stages, the most critical artifact is the prefabricated components. However, in the production stage, the most critical stakeholder is the manufacturer of the prefabricated components; in the transportation stage, the most critical stakeholder is the fleet manager; and in the onsite assembly stage, the most important stakeholder is the main contractor. The status and influence of each stakeholder differ at different stages of the CI supply chain. According to the classical theory of stakeholders, three attributes of stakeholders were used in this research: power to influence the project, urgency of the stakeholder’s claim on the project, and legitimacy of the stakeholder’s relationship to the project. Four attributes of artifacts were also identified, namely, economics, user friendly, social acceptance, and environmental protection. From these seven attributes, the critical attributes for stakeholders and artifacts were identified through a case study. In all three stages, legitimacy is the most important attribute of stakeholders, which is in line with the goals of this study. In the first two stages of production and transportation, the most critical attribute of artifacts is social acceptance, whereas user friendly is the most important attribute of artifacts in the onsite assembly stage. Ineffective logistics, low level of innovation in prefabrication techniques, high initial cost, lack of preferential policy, low degree of standardization, lack of management practices and experiences, and non-timely communication between the parties were considered the key existing issues. Considering the findings of the stages of CI supply chains, stakeholders, artifacts, and their critical attributes, the authors proposed different policy recommendations under a CI backdrop to promote its uptake within the complex socio-technical system.

It is necessary to note the limitations that constrained this study. Although the single case study used in this study was justified as being adequate to obtain significant results through SNA, subsequent studies may improve the number of cases and response rate for better generalization of the results. Moreover, this study is of both theoretical and practical significance. Theoretically, this research offers significant inputs to project management researchers to understand how actants (stakeholders and artifacts) interact in a dynamic, complex socio-technical environment, indicating a new perspective for studies on project management. This research also provides a new direction regarding technology development, mainly based on different types of alliances between stakeholders and artifacts, and the mutually interactive processes of the social and technical aspects. Practically, this study is of value in objectively depicting the real situation of the socio-technical system of CI uptake based on a representative case project in Hong Kong. The outcomes of this research provide valuable implications for the government and policymakers to anticipate the impact of different CI policies on promoting its uptake within the complex socio-technical system.

## Figures and Tables

**Table 1:** Attributes of stakeholders and the artifacts.

**No.**	**Attributes of Stakeholders**	**References**
1	Power to influence the project	[28,29,30,31]
2	Urgency of the stakeholder’s claim on the project	[28,31]
3	Legitimacy of the stakeholder’s relationship with the project	[28,31]
**No.**	**Attributes of the Artifacts**	**References**
1	Economics	[33,34]
2	User friendly	[35,36]
3	Social acceptance	[36,37]
4	Environmental protection	[34,38]

**Table 2:** Main node and sub-node degree centrality vectors of production/transportation/onsite assembly stages.

Stakeholders	2-Mode Normalized Degree Centrality	Artifacts	2-Mode Normalized Degree Centrality
**Production stage**			
HKHS	0.722222	RFID/NFC/QR Code Tag	0.769231
WHSEL	0.944444	Component Code	0.769231
Main Contractor	0.777778	Truck	0.538462
Subcontractors	0.166667	Trailer	0.384615
Logistic Company	0.444444	Gantry Crane	0.461538
Producing Workers	0.777778	Pilot Prefabricated Facades	0.692308
Project Director	1.000000	Prefabricated Facades	0.846154
Managers	0.944444	Contract to confirm production	0.461538
Architect	0.444444	Mater Plan and Design Drawing	0.692308
Testing Organization	0.444444	BIM models	0.538462
Purchasing Staff	0.166667	Prefabrication orders	0.615385
CEO of Manufacturer	1.000000	Delivery/Customer orders	0.769231
QC Inspectors	0.388889	Transportation Plan	0.615385
		Production plan	0.846154
		Production Drawings	0.615385
		Material List	0.461538
		Reinforcement Bars	0.538462
		PPS System	0.769231
**Transportation stage**			
HKHS	0.555556	RFID/NFC/QR Code Tag	1.000000
WHSEL	0.888889	Truck	0.800000
Main Contractor	0.666667	Gantry Crane	0.600000
Logistic Company	1.000000	GPS Device	0.300000
Truck Drivers	1.000000	Prefabricated Facades	1.000000
Fleet Manager	1.000000	Delivery/Customer orders	1.000000
Production Operators	0.777778	Transportation Plan	0.800000
QC Inspectors	0.333333	Loading Lists	0.600000
Production Managers	0.888889	PTS System	0.700000
On-site Operator	0.444444		
**On-site assembly stage**			
HKHS	0.588235	RFID/NFC/QR Code Tag	0.785714
WHSEL	0.647059	Wearable RFID Reader	0.571429
Main Contractor	0.941176	Component Code	0.714286
Logistic Company	0.529412	Truck	0.428571
Subcontractors	0.176471	Tower Crane	0.500000
Truck Drivers	0.529412	Mobile Crane	0.428571
On-site Operator	0.470588	Material Hoist	0.428571
Erection Operators	0.529412	Passenger Hoist	0.428571
Lifting Operators and Supervisor	0.352941	Prefabricated Facades	0.928571
Buffer Operators	0.411765	BIM models	0.357143
Foreman	0.705882	Storage Yard	0.642857
Building Service Inspectors	0.588235	Contract to confirm production	0.357143
Project Manager	0.823529	Master Plan and Design Drawing	0.571429
Engineers	0.352941	Delivery/Customer orders	0.642857
		Transportation Plan	0.428571
		Loading Lists	0.214286
		OAS System	0.857143

**Table 3:** Critical stakeholders and artifacts at production/transportation/onsite assembly stages.

Critical Stakeholders	Critical Artifacts
**Production stage**	
Prefabrication Manufacturer- (WHSEL) CEO of Prefabrication Manufacturer Production Project Director Production Managers Production Workers	Prefabricated Facades Delivery/Customer orders Component Code RFID/NFC/QR Code Tag Production Plan
**Transportation stage**	
Fleet Manager Truck Driver Logistic Company-3PL Prefabrication Manufacturer- (WHSEL) Production Manager	Prefabricated Facades Delivery/Customer orders RFID/NFC/QR Code Tag Truck Transportation Plan
**On-site assembly stage**	
Main Contractor Project Manager HKHS Prefabrication Manufacturer- (WHSEL) Foreman	Prefabricated Facades On-site Assembly Service (OAS) System Component Code RFID/NFC/QR Code Tag Delivery/Customer orders

**Table 4:** Importance of attributes of stakeholders/artifacts at each stage.

	Production		Transportation		Assembly	
	Stakeholders	Artifacts	Stakeholders	Artifacts	Stakeholders	Artifacts
Most important	Legitimacy	Social acceptance	Legitimacy	Social acceptance	Legitimacy	User friendly
Important	Urgency	User friendly	Urgency	User friendly	Urgency Power	Social Acceptance
Less important	Power	Economics	Power	Economics		Economics
Least important		Environmental protection		Environmental protection		Environmental protection

## Data Availability

Data is contained within the article.

## Supplementary Materials

The following are available online at https://www.mdpi.com/article/10.3390/ijerph18179017/s1, Table S1: Relational matrix between stakeholders and artifacts.

## References

[1] Ekanayake E., Shen G.Q., Kumaraswamy M., Owusu E.K. (2020). Critical supply chain vulnerabilities affecting supply chain resilience of industrialized construction in Hong Kong. Eng. Constr. Arch. Manag..

[2] Xu Z., Zayed T., Niu Y. (2020). Comparative analysis of modular construction practices in mainland China, Hong Kong and Singapore. J. Clean. Prod..

[3] Li L., Li Z., Li X., Zhang S., Luo X. (2020). A new framework of industrialized construction in China: Towards on-site industrialization. J. Clean. Prod..

[4] Arif M., Egbu C. (2010). Making a case for offsite construction in China. Eng. Constr. Arch. Manag..

[5] Mak Y.W. (1998). Prefabrication and Industrialization of Housing in Hong Kong. Master’s Thesis.

[6] Luo L., Shen G.Q., Xu G., Liu Y., Wang Y. (2019). Stakeholder-Associated Supply Chain Risks and Their Interactions in a Prefabricated Building Project in Hong Kong. J. Manag. Eng..

[7] HKSAR (2017). The Chief Executive’s 2017 Policy Address.

[8] Seaden G., Manseau A. (2001). Public policy and construction innovation. Build. Res. Inf..

[9] Mao C., Shen Q., Pan W., Ye K. (2015). Major Barriers to Off-Site Construction: The Developer’s Perspective in China. J. Manag. Eng..

[10] Bijker W.E., Hughes T.P., Pinch T.J. (1987). The Social Construction of Technological Systems: New Directions in the Sociology and History of Technology.

[11] Xue J., Shen G.Q., Yang R.J., Zafar I., Ekanayake E., Lin X., Darko A. (2020). Influence of formal and informal stakeholder relationship on megaproject performance: A case of China. Eng. Constr. Arch. Manag..

[12] Geels F. (2004). From sectoral systems of innovation to socio-technical systems: Insights about dynamics and change from sociology and institutional theory. Res. Policy.

[13] Fang D., Chen Y., Wong L. (2006). Safety Climate in Construction Industry: A Case Study in Hong Kong. J. Constr. Eng. Manag..

[14] Dalton T., Hurley J., Gharaie E., Wakefield R., Horne R.E. (2011). Australian Suburban House Building: Industry Organization, Practices and Constraints.

[15] Schiffer E., Hauck J. (2010). Net-map: Collecting social network data and facilitating network learning through participatory influence network mapping. Field Methods.

[16] Meng X. (2012). The effect of relationship management on project performance in construction. Int. J. Proj. Manag..

[17] Zakaria T.S.A.S., Gajendran T., Rose T., Brewer G. (2018). Contextual, structural and behavioural factors influencing the adoption of industrialised building systems: A review. Archit. Eng. Des. Manag..

[18] Gan X., Chang R., Wen T. (2018). Overcoming barriers to off-site construction through engaging stakeholders: A two-mode social network analysis. J. Clean. Prod..

[19] Masood R., Lim J.B., González V.A. (2021). Performance of the supply chains for New Zealand prefabricated house-building. Sustain. Cities Soc..

[20] Luo L.Z., Mao C., Shen L.Y., Li Z.D. (2015). Risk factors affecting practitioners’ attitudes toward the implementation of an industrialized building system a case study from China. Eng. Constr. Archit. Manag..

[21] Wang Y., Li H., Wu Z. (2019). Attitude of the Chinese public toward off-site construction: A text mining study. J. Clean. Prod..

[22] Latour B. (1987). Science in Action: How to Follow Scientists and Engineers through Society.

[23] Freeman R.E. (2010). Two—The Stakeholder Concept and Strategic Management. Strategic Management: A Stakeholder Approach.

[24] Leonardi P.M. (2012). Materiality, Sociomateriality, and Socio-Technical Systems: What Do These Terms Mean? How Are They Different? Do We Need Them?. Mater. Organ..

[25] Ekbia H.R. (2009). Digital artifacts as quasi-objects: Qualification, mediation, and materiality. J. Am. Soc. Inf. Sci. Technol..

[26] Schmidt K., Wagner I. (2004). Ordering systems: Coordinative practices and artifacts in architectural design and planning. Comput. Support. Coop. Work.

[27] Parmaxi A., Zaphiris P., Ioannou A. (2016). Enacting artifact-based activities for social technologies in language learning using a design-based research approach. Comput. Hum. Behav..

[28] Mitchell R.K., Agle B.R., Wood D.J., Mitchell R.K. (1997). Toward a Theory of Stakeholder Identification and Salience: Defining the principle of who and what really counts. Acad. Manag. Rev..

[29] Aaltonen K., Jaakko K., Tuomas O. (2008). Stakeholder salience in global projects. Int. J. Proj. Manag..

[30] Aragonés-Beltrán P., García-Melón M., Montesinos-Valera J. (2017). How to assess stakeholders’ influence in project management? A proposal based on the Analytic Network Process. Int. J. Proj. Manag..

[31] Lin X., Ho C.M.F., Shen G.Q.P. (2017). Who should take the responsibility? Stakeholders’ power over social responsibility issues in construction projects. J. Clean. Prod..

[32] Wood D.J., Mitchell R.K., Agle B.R., Bryan L.M. (2021). Stakeholder identification and salience after 20 years: Progress, problems, and prospects. Bus. Soc..

[33] Chen Y., Okudan G.E., Riley D.R. (2010). Decision support for construction method selection in concrete buildings: Prefabrication adoption and optimization. Autom. Constr..

[34] Safaa Y.P., Hatmoko J.U.D., Purwanggono B. (2019). Evaluation of the use of prefabricated bridge elements with Design for Manufacture and Assembly (DfMA) criteria. MATEC Web Conf..

[35] Lu W., Chen K., Xue F., Pan W. (2018). Searching for an optimal level of prefabrication in construction: An analytical framework. J. Clean. Prod..

[36] Gibb A.G., Isack F. (2003). Re-engineering through pre-assembly: Client expectations and drivers. Build. Res. Inf..

[37] Liu S., Qian S. (2019). Evaluation of social life-cycle performance of buildings: Theoretical framework and impact assessment approach. J. Clean. Prod..

[38] Jaillon L.C., Poon C. (2010). Design issues of using prefabrication in Hong Kong building construction. Constr. Manag. Econ..

[39] Liang X., Shen G.Q., Guo L. (2015). Improving Management of Green Retrofits from a Stakeholder Perspective: A Case Study in China. Int. J. Environ. Res. Public Health.

[40] Yang R.J., Zou P.X.W. (2014). Stakeholder-associated risks and their interactions in complex green building projects: A social network model. Build. Environ..

[41] Li C.Z., Hong J., Xue F., Shen G.Q., Xu X., Mok M.K. (2016). Schedule risks in prefabrication housing production in Hong Kong: A social network analysis. J. Clean. Prod..

[42] Freeman L.C. (1978). Centrality in social networks conceptual clarification. Soc. Netw..

[43] Meghanathan N. (2017). Graph Tools for Social Network Analysis. Graph Theoretic Approaches for Analyzing Large-Scale Social Networks.

[44] Faust K. (1997). Centrality in affiliation networks. Soc. Netw..

[45] Borgatti S.P., Everett M.G. (1997). Network analysis of 2-mode data. Soc. Netw..

[46] Yang R.J., Zou P.X.W., Wang J. (2016). Modelling stakeholder-associated risk networks in green building projects. Int. J. Proj. Manag..

[47] Yu T., Shen G.Q., Shi Q., Lai X., Li C.Z., Xu K. (2017). Managing social risks at the housing demolition stage of urban redevelopment projects: A stakeholder-oriented study using social network analysis. Int. J. Proj. Manag..

[48] Li C.Z., Shen G.Q., Xu X., Xue F., Sommer L., Luo L. (2017). Schedule risk modeling in prefabrication housing production. J. Clean. Prod..

[49] Xu G., Li M., Luo L., Chen C.-H., Huang G.Q. (2018). Cloud-based fleet management for prefabrication transportation. Enterp. Inf. Syst..

[50] Li C.Z. (2017). Integrating RFID and BIM technologies for mitigating risks and improving schedule performance of prefabricated house construction. J. Clean. Prod..

[51] Chiang Y.H., Chan E.H.W., Lok L.K.-L. (2006). Prefabrication and barriers to entry—A case study of public housing and institutional buildings in Hong Kong. Habitat Int..

[52] Li C.Z., Hong J., Xue F., Shen Q., Xu X., Luo L. (2016). SWOT analysis and Internet of Things-enabled platform for prefabrication housing production in Hong Kong. Habitat Int..

[53] Zhou J.X., Shen G.Q., Yoon S.H., Jin X. (2021). Customization of on-site assembly services by integrating the internet of things and BIM technologies in modular integrated construction. Autom. Constr..

[54] Luo L., Jin X., Shen G.Q., Wang Y., Liang X., Li X., Li C.Z. (2020). Supply Chain Management for Prefabricated Building Projects in Hong Kong. J. Manag. Eng..

[55] Slaughter E.S. (2000). Implementation of construction innovations. Build. Res. Inf..

[56] Li L., Li Z., Wu G., Li X. (2018). Critical success factors for project planning and control in prefabrication housing production: A China study. Sustainability.

[57] La Rocca G., Van Tooren M. (2007). Enabling distributed multi-disciplinary design of complex products: A knowledge based engineering approach. J. Des. Res..

[58] Perkins I., Skitmore M., Skitmore R. (2015). Three-dimensional printing in the construction industry: A review. Int. J. Constr. Manag..

[59] Steel J., Drogemuller R., Toth B. (2012). Model interoperability in building information modelling. Softw. Syst. Model..

[60] Bamana F., Lehoux N., Cloutier C. (2019). Simulation of a Construction Project: Assessing Impact of Just-in-Time and Lean Principles. J. Constr. Eng. Manag..

[61] Jiang Y., Zhao D., Wang D., Xing Y. (2019). Sustainable Performance of Buildings through Modular Prefabrication in the Construction Phase: A Comparative Study. Sustainability.

[62] Gao Y., Tian X.-L. (2020). Prefabrication policies and the performance of construction industry in China. J. Clean. Prod..

[63] Lee C., Baldwin A.N. (2008). Reinventing the Hong Kong Construction Industry for Its Sustainable Development.

[64] Wuni I.Y., Shen G.Q. (2020). Barriers to the adoption of modular integrated construction: Systematic review and meta-analysis, integrated conceptual framework, and strategies. J. Clean. Prod..

[65] Jiang W., Luo L., Wu Z., Fei J., Antwi-Afari M.F., Yu T. (2019). An investigation of the effectiveness of prefabrication incentive policies in China. Sustainability.

[66] HKBD (2001). Joint Practice Note No. 1, Green and Innovative Buildings.

[67] Gan X., Chang R., Zuo J., Wen T., Zillante G. (2018). Barriers to the transition towards off-site construction in China: An Interpretive structural modeling approach. J. Clean. Prod..

[68] Jiang W., Huang Z., Peng Y., Fang Y., Cao Y. (2020). Factors affecting prefabricated construction promotion in China: A structural equation modeling approach. PLoS ONE.

[69] Gan X.L., Chang R.D., Langston C., Wen T. (2019). Exploring the interactions among factors impeding the diffusion of prefabricated building technologies: Fuzzy cognitive maps. Eng. Constr. Archit. Manag..

[70] Building Department (2016). Code of Practice for Precast Concrete Construction.

[71] Si T., Li H.X., Hosseini M.R., Ji Y., Liu C. A Solution to Just-in-Time Delivery for Off-Site Construction: A Conceptual Model. Proceedings of the Construction Research Congress (CRC) on Construction Research and Innovation to Transform Society.

[72] Jiang L., Li Z., Li L., Gao Y. (2018). Constraints on the Promotion of Prefabricated Construction in China. Sustainability.

[73] Xu X., Xiao B., Li C.Z. (2021). Stakeholders’ power over the impact issues of building energy performance gap: A two-mode social network analysis. J. Clean. Prod..

[74] Li X., Chi H.L., Wu P., Shen G.Q. (2020). Smart work packaging-enabled constraint-free path re-planning for tower crane in prefabricated products assembly process. Adv. Eng. Inform..

[75] Li X., Wu L., Zhao R., Lu W., Xue F. (2021). Two-layer Adaptive Blockchain-based Supervision model for off-site modular housing production. Comput. Ind..

[76] Ekanayake E.M.A.C., Shen G., Kumaraswamy M.M. (2021). Identifying supply chain capabilities of construction firms in industrialized construction. Prod. Plan. Control.

