# Supplementary material for: Improving Construction Industrialization Practices from a Socio-Technical System Perspective: A Hong Kong Case
Source: Int J Environ Res Public Health. 2021 Aug 26;18(17):9017. doi: 10.3390/ijerph18179017 (PMC8431180; doi:10.3390/ijerph18179017)
Supplement: Supplementary file 1 [file ijerph-18-09017-s001.zip › ijerph-1327002-supplementary.pdf]

Table S1: Relational matrix between stakeholders and artifacts

Production Stage

Artifact / Stakeholder	RFID/NFC/QR Code Tag	Component Code	Truck	Trailer	Gantry Crane	Pilot Prefabricated Facades	Prefabricated Facades	Contract to confirm prefabrication production	Mater Plan & Design Drawing	BIM models	Prefabrication orders	Delivery/Customer orders	Transportation Plan	Production plan	Prefabrication Production Drawings	Material List	Reinforcement Bars	Prefabrication Production Service (PPS) System
Hong Kong Housing Society (HKHS)	1	1	0	0	0	1	1	1	1	1	1	1	1	1	1	0	0	1
Prefabrication Manufacturer-Wing Hong Shun Enterprise Limited (WHSEL)	1	1	1	1	1	1	1	1	1	0	1	1	1	1	1	1	1	1
Main Contractor - Aggressive Construction Co., LTD.	1	1	1	0	0	1	1	1	1	1	1	1	1	1	1	0	0	1
Subcontractors	0	0	0	0	0	0	0	0	1	1	0	0	0	1	0	0	0	0
Logistic Company-3PL	1	1	1	0	1	0	1	0	0	0	0	1	1	0	0	0	0	1
Prefabrication Producing Workers	1	1	1	1	1	1	1	0	1	0	1	1	1	1	1	0	1	0
Production Project Director	1	1	1	1	1	1	1	1	1	1	1	1	1	1	1	1	1	1
Production Managers	1	1	1	1	1	1	1	0	1	1	1	1	1	1	1	1	1	1
HKHS Contract Manager (Architect)	0	0	0	0	0	0	1	1	1	1	1	1	0	0	1	0	0	1
Testing Organization	1	1	0	0	0	1	1	0	0	0	0	0	0	1	0	1	1	1
Purchasing Staff	0	0	0	0	0	0	0	0	0	0	0	0	0	1	0	1	1	0
CEO of Prefabrication Manufacturer	1	1	1	1	1	1	1	1	1	1	1	1	1	1	1	1	1	1
Quality Control Inspectors	1	1	0	0	0	1	1	0	0	0	0	1	0	1	0	0	0	1

Transportation stage

Stakeholder \ Artifact	RFID/NFC/QR Code Tag	Truck	Gantry Crane	GPS Device	Prefabricated Facades	Delivery/Customer orders	Transportation Plan	Loading Lists	Prefabrication Transportation Service (PTS) System
Hong Kong Housing Society (HKHS)	1	0	0	0	1	1	1	0	1
Prefabrication Manufacturer- Wing Hong Shun Enterprise Limited (WHSEL)	1	1	1	0	1	1	1	1	1
Main Contractor - Aggressive Construction Co., LTD.	1	1	0	0	1	1	1	0	1
Logistic Company-3PL	1	1	1	1	1	1	1	1	1
Truck Drivers	1	1	1	1	1	1	1	1	1
Fleet Manager	1	1	1	1	1	1	1	1	1
Production Operators	1	1	1	0	1	1	1	1	0
Quality Control Inspectors	1	0	0	0	1	1	0	0	0
Production Managers	1	1	1	0	1	1	1	1	1
On-site Operator	1	1	0	0	1	1	0	0	0

On-site assembly stage

Artifact / Stakeholder	RFID/NFC/QR Code Tag	Wearable RFID Reader	Component Code	Truck	Tower Crane	Mobile Crane	Material Hoist	Passenger Hoist	Prefabricated Facades	BIM models	Prefabricated Facades Storage Yard	Contract to confirm prefabrication production	Mater Plan & Design Drawing	Delivery/Customer orders	Transportation Plan	Loading Lists	On-site Assembly Service (OAS) System
Hong Kong Housing Society (HKHS)	1	0	1	0	0	0	0	0	1	1	1	1	1	1	1	0	1
Prefabrication Manufacturer-Wing Hong Shun Enterprise Limited (WHSEL)	1	1	1	1	0	0	0	0	1	0	0	1	1	1	1	1	1
Main Contractor - Aggressive Construction Co., LTD.	1	1	1	1	1	1	1	1	1	1	1	1	1	1	1	0	1
Logistic Company-3PL	1	1	1	1	0	0	0	0	1	0	0	0	0	1	1	1	1
Subcontractors	0	0	0	0	0	0	0	0	0	1	1	0	1	0	0	0	0
Truck Drivers	1	1	0	1	0	1	0	0	1	0	0	0	0	1	1	1	1
On-site Operator	1	1	0	1	0	1	0	0	1	0	1	0	0	1	0	0	1
Erection Operators	1	1	1	0	1	0	1	1	1	0	0	0	1	0	0	0	1
Lifting Operators & Supervisor	0	0	1	0	1	0	1	1	1	0	1	0	0	0	0	0	0
Buffer Operators	1	1	1	0	0	0	0	0	1	0	1	0	0	1	0	0	1
Foreman	1	0	1	1	1	1	1	1	1	0	1	0	1	1	0	0	1
Building Service Inspectors	1	1	1	0	1	1	1	1	1	0	1	0	0	0	0	0	1
On-site Project Manager	1	0	1	0	1	1	1	1	1	1	1	1	1	1	1	0	1
On-site Engineers	0	0	0	0	1	0	0	0	1	1	0	1	1	0	0	0	1
